# X-Ray Induced Formation of γ-H2AX Foci after Full-Field Digital Mammography and Digital Breast-Tomosynthesis

**DOI:** 10.1371/journal.pone.0070660

**Published:** 2013-07-25

**Authors:** Siegfried A. Schwab, Michael Brand, Ina-Kristin Schlude, Wolfgang Wuest, Martina Meier-Meitinger, Luitpold Distel, Ruediger Schulz-Wendtland, Michael Uder, Michael A. Kuefner

**Affiliations:** 1 Institute of Radiology, University Erlangen/Nuremberg, Erlangen, Bavaria, Germany; 2 Department of Radiation Oncology, University Erlangen/Nuremberg, Erlangen, Bavaria, Germany; Kagoshima University Graduate School of Medical and Dental Sciences, Japan

## Abstract

**Purpose:**

To determine in-vivo formation of x-ray induced γ-H2AX foci in systemic blood lymphocytes of patients undergoing full-field digital mammography (FFDM) and to estimate foci after FFDM and digital breast-tomosynthesis (DBT) using a biological phantom model.

**Materials and Methods:**

The study complies with the Declaration of Helsinki and was performed following approval by the ethic committee of the University of Erlangen-Nuremberg. Written informed consent was obtained from every patient. For in-vivo tests, systemic blood lymphocytes were obtained from 20 patients before and after FFDM. In order to compare in-vivo post-exposure with pre-exposure foci levels, the Wilcoxon matched pairs test was used. For in-vitro experiments, isolated blood lymphocytes from healthy volunteers were irradiated at skin and glandular level of a porcine breast using FFDM and DBT. Cells were stained against the phosphorylated histone variant γ-H2AX, and foci representing distinct DNA damages were quantified.

**Results:**

Median in-vivo foci level/cell was 0.086 (range 0.067–0.116) before and 0.094 (0.076–0.126) after FFDM (p = 0.0004). In the in-vitro model, the median x-ray induced foci level/cell after FFDM was 0.120 (range 0.086–0.140) at skin level and 0.035 (range 0.030–0.050) at glandular level. After DBT, the median x-ray induced foci level/cell was 0.061 (range 0.040–0.081) at skin level and 0.015 (range 0.006–0.020) at glandular level.

**Conclusion:**

In patients, mammography induces a slight but significant increase of γ-H2AX foci in systemic blood lymphocytes. The introduced biological phantom model is suitable for the estimation of x-ray induced DNA damages in breast tissue in different breast imaging techniques.

## Introduction

Besides ultrasound and magnetic resonance imaging (MRI), conventional x-ray mammography is one of the most valuable diagnostic tools for imaging of the breast. Traditionally performed as a film-based method, digital techniques as full-field digital mammography (FFDM) are primarily in use nowadays. The continuous technical development led to the introduction of new imaging techniques like digital breast tomosynthesis (DBT) [1], [2], phase contrast X-ray imaging [3] or computed tomography of the breast [4]. Despite the technical innovations, all these methods still require exposure of breast tissue to ionizing radiation, and it is becoming more and more difficult to compare theses imaging modalities in regard to their radiation dose. For an estimation of the delivered doses, physical measurements with ionization chambers and mathematical operations and registration of exposure parameters are in use [5]. However, while these parameters determine radiation dose, they are not able to assess biological x-ray interactions in the body of the patient. Recently, a new technique for the detection and quantification of in-vivo DNA-damages has been developed. DNA double-strand breaks (DSBs) are regarded as the most relevant DNA lesions induced by ionizing irradiation. One of the first answers of the cell after induction of DSBs is the phosphorylation of the histone variant H2AX. The phosphorylated histones (named γ-H2AX) can be monitored as distinct cytologically visible “foci” using a specific antibody and fluorescence microscopy [6]. This method has been shown to be a reliable and sensitive tool for the determination of DNA lesions introduced by computed tomography (CT) or conventional angiography [7]–[10]. However, compared to these modalities, in breast imaging the radiation dose is quite lower and the quality of the x-rays is different due to lower tube voltage, different filtration and dedicated anode materials. Additionally, the female breast is considered as a rather radiosensitive organ.

Therefore, the purpose of our research was to determine whether the γ-H2AX immunoflourescence method is suitable for the measurement of x-ray induced DNA damages in different X-ray based breast imaging techniques, like FFDM and DBT.

## Materials and Methods

### Validation of foci quantification

For the validation of the immunofluorescence method used in this study, we performed in-vitro experiments. After obtaining written informed consent, blood samples were taken from three female healthy volunteers (age 26–34 years) using EDTA containing plastic vials (S-Monovette, Sarstedt, Nümbrecht, Germany). For generation of dose effect curves, these samples (n = 3) were irradiated at room temperature with 5, 10, and 50 mGy and incubated for 5 minutes at standard conditions (37°C, 5% CO2, 95% air).

In order to evaluate a time plot of foci levels, samples pre-treated with 1 nM calyculin A (which is a potent phosphatase inhibitor and prevents from foci loss) were irradiated with 50 mGy and incubated for 0, 1, 5, 10, 15, and 30 minutes (n = 3). Further work-up up of the samples was performed as described below.

### Patients, mammographies, and in-vivo samples

The study complies with the Declaration of Helsinki and was performed following approval by the ethic committee of the University of Erlangen-Nuremberg. On days where manpower in the laboratory was available, 20 consecutive females were included (median age 54 years, range 39–71 years), who underwent FFDM for the routine diagnostic work up of unclear breast nodules, follow-up of benign disease or routine screening. All of the mammographies were performed due to clinical indications – no additional x-ray exposure was performed for the study. Written informed consent was obtained from every patient. Criteria to be excluded from the study were radiation therapy or chemotherapy within the last six months, x-ray examination within the last three days, and history of lymphoma, leukaemia or other malignant tumors. These are exclusion criteria which were used in various previous studies evaluating in-vivo γ-H2AX foci formation after radiologic examinations [7], [8], [11], [12].

FFDM was performed in a craniocaudal and a medial lateral oblique view of each breast (resulting in 4 images in each patient), using a digital full-field system based on an anode/filter combination of tungsten/rhodium using automated dose regulation (MAMMOMAT Inspiration, Siemens Healthcare, Forchheim, Germany). None of the patients underwent additional x-ray exposure like DBT. Blood samples were taken from the antecubital vein before and 5 minutes after the exposure, using ethylenediaminetetraacetic acid (EDTA) containing vials (S-Monovette, Sarstedt, Nümbrecht, Germany) and stored at 4°C during transport to the laboratory. This time point was chosen since highest γ-H2AX values were obtained in blood lymphocytes 5 minutes after radiation exposure [13], [14]. In order to prevent a foci loss by DNA repair, 1 nM Calyculin A - which is a potent inhibitor of the dephosphorylation of γ-H2AX - was added to each vial [15].

The imaging parameters of the mammograms (compression thickness, peak kilovoltage (kVp), milliampere-seconds (mAs)) as well as the exposure data (entrance dose including backscatter and glandular dose) were registered for all patients as indicated by the digital full-field system (Table 1). Correctness of dose recordings by the mammography unit is verified by monthly dosimetry. No deviation of more than 10% is accepted.

**Table 1:** Patients with two view mammography of both breasts: patient's age in years (age), mean compression thickness in cm (thickness), mean tube current time product in mAs (mAs), mean tube voltage in kV (kVp), total entrance dose of four views in mGy (ED), total glandular dose of four views in mGy (GD).

Patient No.	age	Thickness	mAs	kVp	ED	GD
1	57	60.8	91.4	30.0	14.1	4.2
2	49	65.5	153.0	30.0	24.5	6.7
3	61	52.8	74.3	29.0	10.3	3.5
4	67	52.0	110.0	28.8	14.7	5.3
5	71	61.5	95.1	29.8	14.5	4.3
6	50	63.3	110.0	30.0	17.2	4.9
7	58	46.3	106.0	28.3	13.4	5.1
8	42	49.5	109.0	28.5	14.2	5.1
9	51	47.0	109.0	28.3	13.7	5.3
10	60	54.5	105.0	29.3	14.9	4.9
11	41	50.3	93.3	28.8	12.4	4.4
12	39	40.3	58.6	28.0	7.1	3.1
13	55	47.3	96.7	28.0	12.0	4.5
14	45	40.3	98.3	27.8	11.5	5.0
15	61	54.3	83.5	29.0	11.6	4.0
16	52	69.8	244.0	30.8	41.1	10.8
17	54	38.5	65.6	27.5	7.4	3.3
18	61	53.0	76.2	29.0	10.6	3.7
19	46	59.5	214.0	29.5	31.8	9.7
20	70	62.8	150.0	30.0	23.3	6.6
**median**	**54.5**	**52.9**	**102.0**	**29.0**	**13.9**	**4.9**

### Biological phantom model

**Figure 1:**
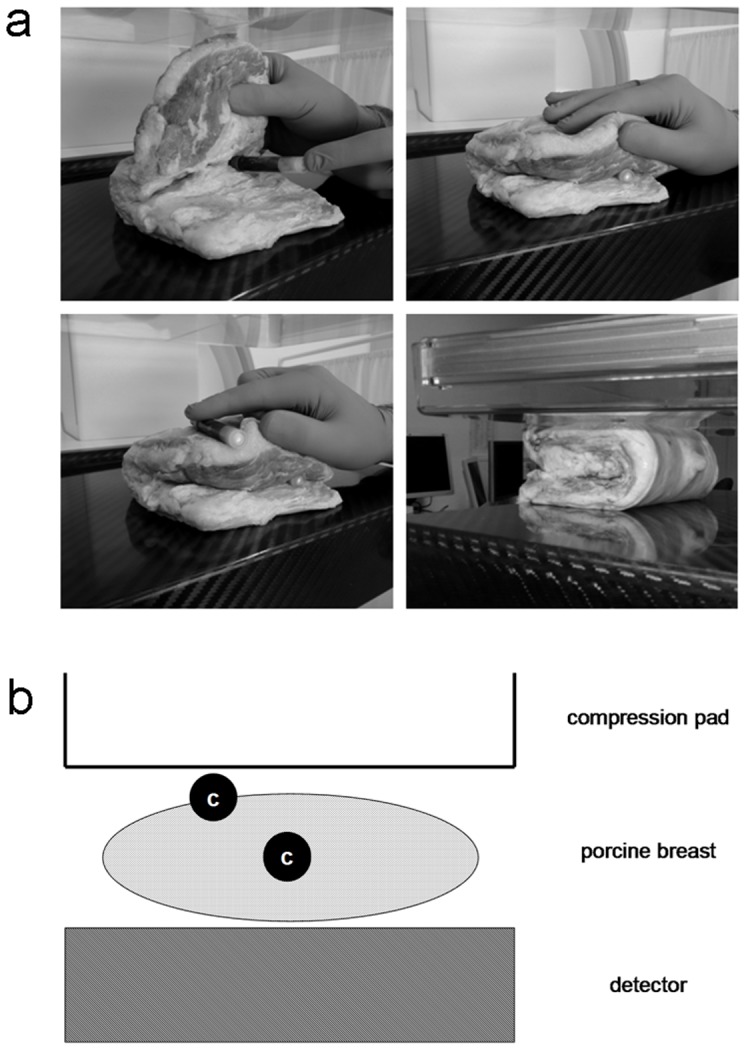
Setup for in-vitro testing (photographs (a), scheme (b)). A cadaveric porcine breast simulates the human breast. Blood containing vials (c in scheme) were exposed to x-rays at skin and glandular level using a commercially available dedicated breast imaging unit.

For the biological phantom model, the human female breast was simulated by a porcine cadaveric breast. Blood samples were obtained from ten healthy individuals (age 26–34 years). Blood containing plastic vials (S-Monovette, Sarstedt, Nümbrecht, Germany) were located in the center of the porcine breast to simulate glandular exposure, or on the surface to simulate the skin exposure (figure 1). It is important to use plastic vials for irradiation since glass can increase radiation induced γ-H2AX foci formation due to photoelectrons arising in the glass material [16]. The porcine breast was compressed to a thickness of 40 mm for all experiments. For the simulation of FFDM and DBT, the same digital full-field system as for the in-vivo mammographies (MAMMOMAT Inspiration, Siemens Healthcare, Germany) was used. All exams were performed with a preselected anode/filter combination of tungsten/rhodium at 80 mAs and 30 kVp without automated dose regulation. To simulate clinical conditions of FFDM with mammograms in two views each sample was irradiated twice at room temperature. For the estimation of foci after DBT, the samples were irradiated once, using the automated tomosynthesis mode in which the x-ray tube moves along a limited arc allowing multiple low-dose images to be acquired. 1 nM calyculin A was added to each sample before exposure. Each experiment was performed 10 times. Non-irradiated blood samples were used as controls.

The exposure data (entrance dose and glandular dose) were registered for experiments as indicated by the digital full-field system.

### γ-H2AX immunofluorescence microscopy

Lymphocytes were isolated and stained as described before [9], [10]. Blood samples were diluted 1∶2 with RPMI medium 1640 (Biochrom, Berlin, Germany) supplemented with 10% fetal calf serum (FCS) and penicillin/streptomycin (50 U/ml and 50 µg/ml, respectively). For the separation of lymphocytes 6 ml of diluted blood was layered onto 6 ml of lymphocyte separation medium 1077 (Biochrom, Berlin, Germany), and centrifuged at 1200×g for 15 minutes (37°C). Lymphocytes from the interphase were washed for 10 minutes in RPMI medium 1640 supplemented with 10% FCS and penicillin/streptomycin (50 U/ml and 50 µg/ml, respectively) and in phosphate buffered saline (PBS, pH 7.0) at 37°C, respectively. This separation yields approximately 80% lymphocytes, 15% monocytes and 5% granulocytes, as described before [9]. Cells were resuspended in 200 µl PBS and spotted onto microscope slides for ten minutes at room temperature, followed by fixation in 100% methanol (20 min, −20°C) and permeabilization in 100% acetone (1 min, −20°C). Each sample was then washed in PBS/1% FCS for 3×10 minutes at room temperature. Staining was performed using a specific γ-H2AX antibody (Anti-H2A.X-Phosphorylated (Ser 139), BioLegend, Uithoorn, The Netherlands) at a dilution of 1∶2500 at 4°C overnight. The next day they were washed twice in PBS/1% FCS, once in PBS, stored for 20 minutes in 2.5% formaldehyde, and washed three times in PBS/1% FCS prior to incubation with Alexa Fluor 488-conjugated goat anti-mouse secondary antibody (Invitrogen, Paisley, UK) at a dilution of 1∶400 for one hour at room temperature. Lymphocytes were washed four times in PBS pH 7.0 at room temperature and mounted with VECTASHIELD© mounting medium containing 4′,6-diamidino-2-phenylindole (Vector Laboratories, Burlingame, USA).

Fluorescence analyses were performed in a blinded fashion using a DM 6000 B microscope (Leica, Wetzlar, Germany) equipped with a ×100 magnification objective. In each sample as many cells were counted until 40 γ-H2AX foci were detected and the foci numbers were related to the number of enumerated cells. This approach is well established in foci quantification and has been described by various authors [6], [9], [17]. Numbers of excess foci were calculated by subtracting the foci number of corresponding pre-exposure from the post-exposure samples obtained after in-vitro or in-vivo irradiation and represent the numbers of x-ray induced DNA damages.

### Statistical analysis

In order to compare in-vivo post-exposure with pre-exposure foci levels, the Wilcoxon matched pairs test was used. For assessment of the correlation of the dose effect curve, Spearman correlation was calculated. The tests were two-sided. For analysis of the in-vitro time plot, repeated measures test was performed. A p-value <0.05 was considered statistically significant. Statistical analysis was performed using the software Prism 4.03, 2005 (Graph-Pad Software, San Diego, CA).

## Results

### Validation of foci quantification

**Figure 2:**
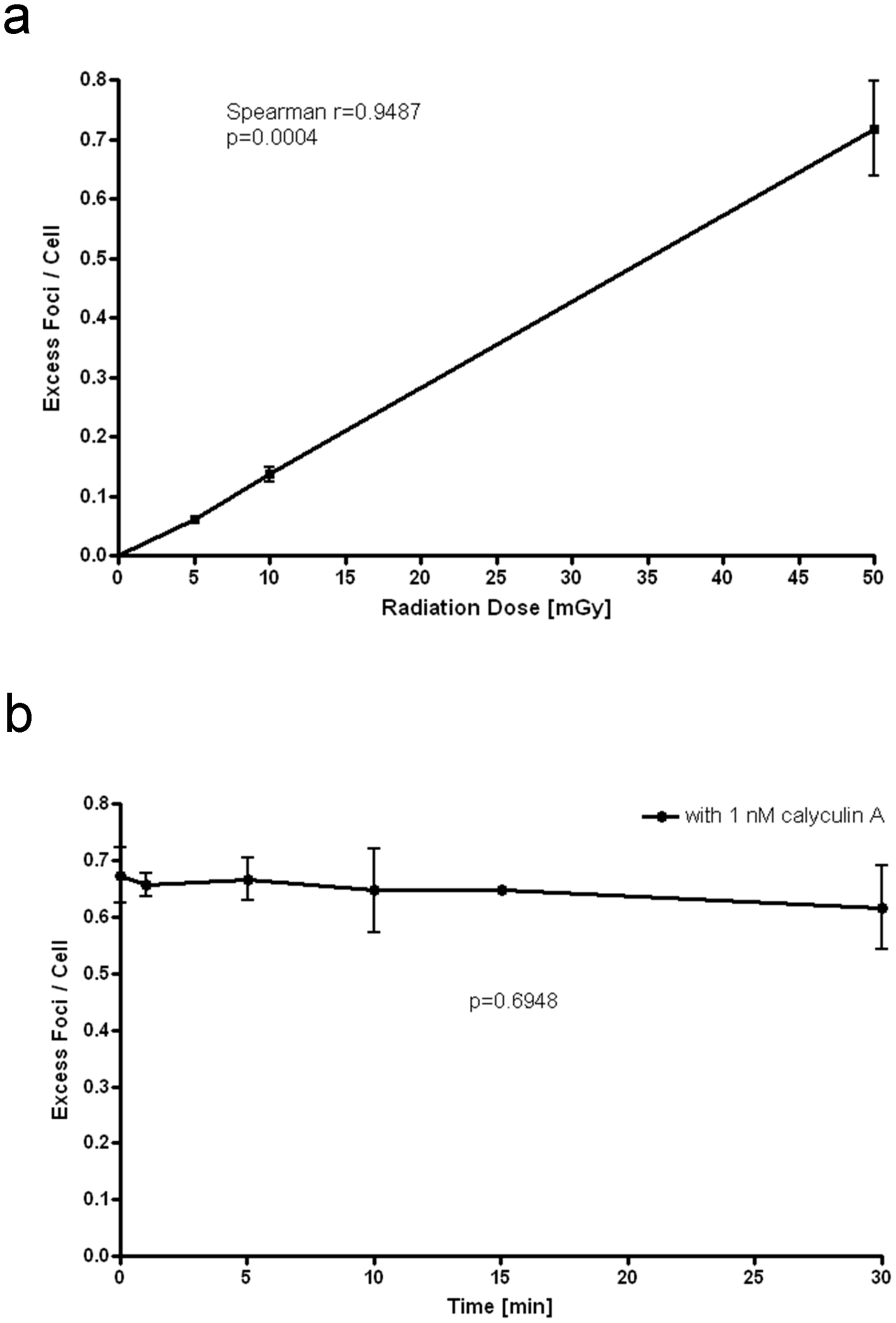
Validation of γ-H2AX foci quantification. (a) illustrates the dose effect curve. Samples were irradiated with doses ranging from 5 to 50 mGy and were incubated for 5 minutes (n = 3). (b) shows a time plot of foci in lymphocytes pre-treated with 1 nM Calyculin A after irradiation with 50 mGy (n = 3). Means and standard deviations are presented. P<0.05 was considered statistically significant.

The in-vitro generated dose effect curve showed a linear correlation between γ-H2AX foci levels and the radiation dose for a dose range between 5 and 50 mGy (Spearman r = 0.9487, p = 0.0004; figure 2a).

The time plot of foci levels in lymphocytes pre-treated with 1 nM calyculin A did not show a significant difference between values at different time points (p = 0.6948) and therefore no relevant foci loss within 30 minutes after irradiation (figure 2b).

### Patients, mammographies, and in-vivo samples

None of our patients had a history of breast cancer. The reasons for performing routine FFDM were work up of unclear breast nodules (n = 3), mastodynia (n = 2), follow-up of BIRADS 3 lesions (n = 9) or routine screening (n = 6). The mammographies of 40 breasts in 20 patiens were classified as BIRADS 1/2 in 36 cases, BIRADS 3 in 3 breasts and BIRADS 5 in one case. 2 of the 3 lesions classified as BIRADS 3 did not show any morphological changes in the follow-up examinations and were therefore classified as BIRADS 2. One patient with the BIRADS 3 lesion did not undergo the suggested follow-up examination. The patient with the BIRADS 5 lesion underwent ultrasound guided core biopsy, histology showed an invasive-ductal carcinoma. Table 1 shows patientś age, the mean compression thickness, mean mAs, mean kVp, total entrance dose and total glandular dose of the two view mammographies of both breasts.

**Figure 3:**
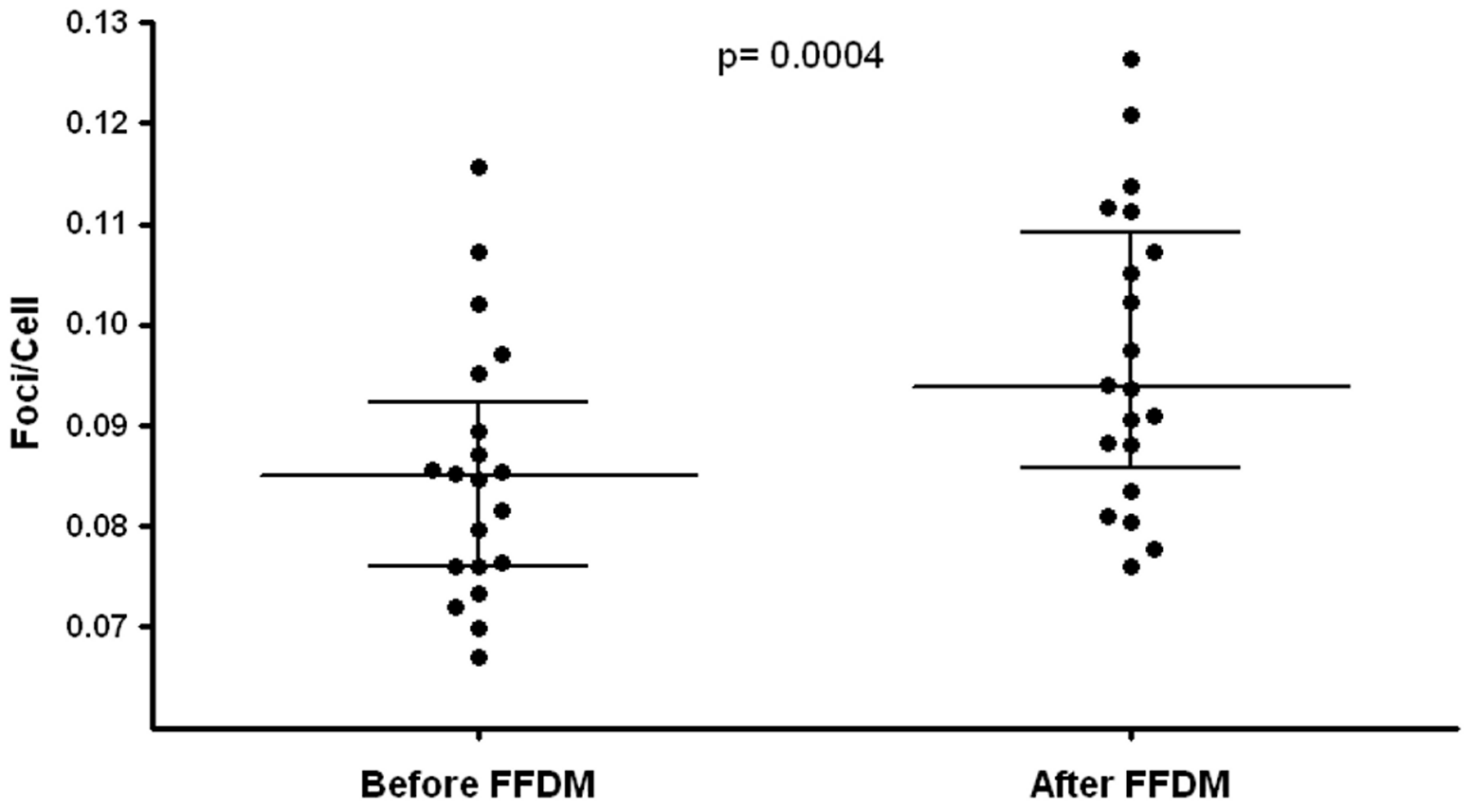
In-vivo γ-H2AX foci/cell representing DNA damages in systemic blood lymphocytes of patients (n = 20) before (left) and after (right) two view full field digital mammography (FFDM) of both breasts. Dots show individual values of each patient, the middle horizontal line represents the median, and the error bars indicate the interquartile ranges. A p value is shown, p<0.05 was considered statistically significant.

The number of analyzed cells for each data point was dependent on the number of foci. The ranges of counted cells for quantification of γ-H2AX foci were 345–597 for pre-exposure and 317–526 for post-exposure in-vivo samples. The median baseline level of foci per cell was 0.086 (range 0.067–0.116). The median post-exposure in-vivo level was 0.094 (0.076–0.126), resulting in a significant increase of median excess foci per cell of 0.008 (0.001–0.026; p<0.0004), as illustrated in figure 3.

### Biological phantom model

For FFDM, the entrance and glandular dose as provided by the imaging unit was 5.80 (two views, each 2.90) and 2.54 (two views, each 1.27) mGy respectively, for DBT it was 2.90 and 1.19 mGy.

The ranges of counted cells for quantification of in-vitro γ-H2AX foci were 625–800 (baseline levels), 200–278 (FFDM skin level), 384–439 (FFDM glandular level), 289–384 (DBT skin level), and 512–588 (DBT glandular level).

**Figure 4:**
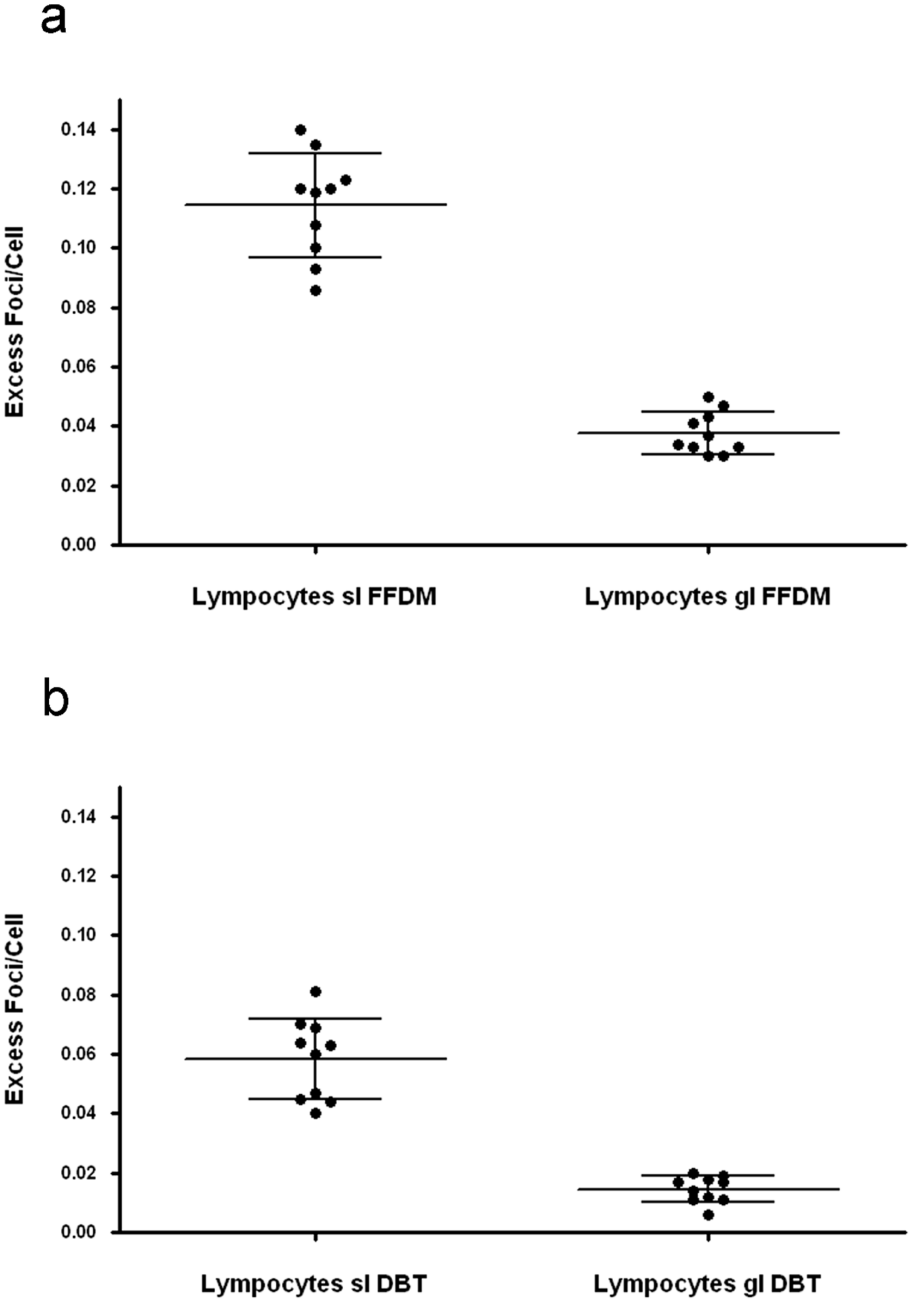
In-vitro γ-H2AX excess foci per cell representing x-ray induced DNA damages in blood lymphocytes after a) full-field digital mammography (FFDM) and b) digital breast tomosynthesis (DBT) at glandular (gl) and skin level (sl), respectively. Dots show individual data of 10 independent experiments, the middle horizontal lines represent means and the error bars represent standard deviations.

The median number of γ-H2AX foci/cell in blood lymphocytes before exposure was 0.057 (range 0.050–0.064), and served as baseline levels. After FFDM, the median post-exposure level was 0.177 (range 0.144–0.198) at skin level and 0.095 foci/cell (range 0.091–0.104) at glandular level. The resulting median excess foci/cell was 0.120 (range 0.086–0.140) at skin level, whereas it was 0.035 (range 0.030–0.050) at glandular level. After DBT, the median foci/cell was 0.119 (range 0.104–0.138) at skin level and 0.075 (range 0.068–0.078) at glandular level. The resulting median excess foci/cell was 0.061 (range 0.040–0.081) at skin level, whereas it was 0.015 (range 0.006–0.020) at glandular level. The distinct values of all experiments are demonstrated in figure 4.

## Discussion

In our study a slight albeit significant elevation of x-ray induced γ-H2AX foci was obtained in systemic blood lymphocytes of women undergoing mammographies. However, using a biological phantom model, a clear induction of foci was detectable in lymphocytes both after FFDM and DBT too. The foci damage level was higher after FFDM compared to DBT.

The method used in this study is based on the early phosphorylation of the histone variant H2AX [18]. Distinct foci can be visualized within the cells using fluorescence microscopy. A close correlation between γ-H2AX foci and DNA DSBs have been observed previously by comparing number of γ-H2AX foci with DNA damages obtained by pulsed-field gel electrophoresis, which is an established method for the determination of DSBs but works only after exposure to higher x-ray doses [6]. However, foci formation can also occur at single-stranded DNA regions or during normal replication. The use of non-replicating cells like lymphocytes used in our study can be helpful to avoid this problem [19], [20]. Despite some limitations, the assessment of γ-H2AX foci formation and loss can be used to evaluate DSB induction and repair [19], [21].

γ-H2AX immunofluorescence microscopy is able to detect radiation induced γ-H2AX foci after irradiation with doses less than 1 mGy in-vitro and in-vivo, and the damage levels obtained correlate with the x-ray dose deposed [7]–[10], [22].

In previous studies, in-vivo induction of γ-H2AX foci was only detected in systemic blood lymphocytes of patients undergoing examinations using higher doses as CT and angiography. Despite the low radiation dose of FFDM, our presented results provide a slight but significant in-vivo increase of γ-H2AX foci after mammography compared to the controls obtained before exposure. These results indicate the high sensitivity of γ-H2AX immunofluorescence microscopy technique used in our experiments. However, the female breast has a considerably lower blood volume than abdominal organs or the heart, which reduces the quantity of exposed blood lymphocytes [7], [8], [10], [23]. Additionally the compression of the breast may lead to an expression of blood out of the breast tissue and thus again may reduce the amount of irradiated blood cells at mammography. Moreover, there is an effect of the dilution of the x-ray exposed lymphocytes by non-radiated blood.

To overcome these limitations and to simulate in-vivo conditions, we established an in-vitro phantom model using a porcine breast and blood lymphocytes for the estimation of the local DNA damage of cells within the breast. We chose to use blood lymphocytes of young healthy individuals, as the determination of γ-H2AX foci is well established in these cells [7]–[10], [23]. Normally γ-H2AX foci disappear over time due to DNA repair. This does not affect our in-vitro results since calyculin A prevents foci loss as shown in figure 2b. It has to be mentioned at this point, that, for in-vitro testing, calyculin A was added prior to exposure, while for obvious reasons it was added following exposure for in-vivo testing, which may lead to a certain bias in regard to comparison of in-vitro with in-vivo formation of γ-H2AX foci. Therefore, the comparison was not carried out in this study. The comparability of in-vitro and in-vivo results may be further constrained because of differences in blood temperature at the time of irradiation, which was room temperature for in-vitro experiments and body temperature for in-vivo tests. Since, in mammography, the entrance dose and the glandular dose are different, we evaluated the biological x-ray effects at the skin on the surface and at the glandular level in the center of the breast [5], [24]. 

Corresponding to the dose estimation which was indicated by the digital full-field system, the damage level was higher at the skin level than inside the porcine breast, which can be explained by the absorption of the x-rays in the breast tissue.

Digital breast tomosynthesis is a novel technology for breast imaging, which is based on three-dimensional reconstructions of multiple low-dose single images which are acquired in different projections, and so far only limited data about its dose aspects exist [5]. In our study, we could find an increase in γ-H2AX foci both at skin and glandular level. The measured foci from a single DBT were around one third up to a half of the foci levels resulting from the two view mammography, which correlates well with the exposure values (entrance dose and glandular dose) provided by the imaging unit. Therefore our model seems to be suitable to compare x-ray induced DNA damages between various breast imaging techniques and can also be used for the determination of biological radiation effects in novel modalities (e.g. breast CT).

Previous studies tried to simulate cellular damage in human fibroblasts, human hybrid cells or human lymphocytes due to mammography-like in-vitro irradiation, analysing chromosomal aberrations, cellular mutation analyses and even DSBs by pulsed-field gel electrophoresis [25]–[28]. In none of these preceding examinations commercially available mammography systems have been used. Colin et al. [29], [30] used γ-H2AX foci as an indicator for DNA damage in breast cells which were cultivated from breast biopsies and irradiated the samples in a mammography system. In another recent study, fibroblasts of BRCA1 and BRCA2 mutation carriers, a BRCA2-deficient fanconi anemia patient and normal individuals were exposed to mammography X-rays and chromosomal anomalies were assessed [31]. However, in contrast to our experiments, in these studies no phantom model was used to simulate the female breast and no in-vivo data were obtained.

Although our model using a porcine cadaveric breast is a cheap and easily achievable approach which can be used as an alternative to complicated and/or expensive breast phantoms and even though γ-H2AX immunofluorescence microscopy is a reliable and sensitive technique for the determination of x-ray induced DNA damages, there are some limitations which must be discussed. It remains unclear, to which extent medication or diseases in our study subjects might have influenced the level of γ-H2AX foci in the in-vivo testing. Possibly an ideal collective of individuals would have consisted of healthy women without any known medication. However, many women undergoing mammography suffer from other diseases, and thus our study subjects reflect normal clinical situation. In addition our exclusion criteria have been established in various previous studies evaluating in-vivo γ-H2AX foci formation after radiologic examinations, since these criteria address the most important influence factors for foci quantification in lymphocytes [7], [8], [11], [12].

As mentioned above, in-vivo and in-vitro testing was performed under slightly different conditions (temperature, time point of addition of calyculin A) and therefore a direct comparison of DNA effects is not adequately achievable. As most probably the intramammary blood volume changes with breast size, a correlation between the frequency of foci/cells in systemic blood lymphocytes and the volume of the breast seems probable. However, as our patient population was rather small (n = 20), we considered splitting it up into more sub-populations was not going to produce representative results.

In our phantom model we did not use mammary cells for several reasons. Tumour cell lines can express higher endogenous amounts of γ-H2AX. Micronucleus formation, alternations in chromatin structures and normal replication can lead to γ-H2AX foci formation in replicating cells. Therefore we used lymphocytes - which are non-replicating cells - in order to avoid the above mentioned confounding factors.

So it must be kept in mind, that the in-vitro experiments represent only a model to simulate the in-vivo reality and quantifying γ-H2AX foci in blood lymphocytes reflects only indirectly the local effects of radiation in the breast. However, taking tissue specimen out of a human breast before and after breast imaging for scientific reasons is not possible due to ethical reasons and the biopsy risk for the patient. However, various tissues seem to react in a similar fashion to in-vivo iradiation, as shown before [32]. Therefore, we believe that our in-vitro results represent a good estimation of the DNA damages in breast tissue caused by FFDM and DBT. Finally our results do not provide evidence about the cancer risk of mammography or tomosynthesis since the misrepair rate of DNA damages remains unclear.

## Conclusions

γ-H2AX immunofluorescence microscopy represents a very sensitive method for the determination of x-ray induced DNA damages. Even after mammography, a slight but significantly measurable induction of γ-H2AX foci levels in systemic blood lymphocytes of patients can be detected. Using the biological phantom model based on the γ-H2AX immunofluorescence method, a clear induction of DNA lesions both by FFDM and DBT can be found, too. Therefore this model is suitable for the estimation of x-ray induced DNA damages in breast tissue and can be used to compare different breast imaging techniques relating to their DNA damaging x-ray effects.
